# Acute Kidney Injury Following the Ingestion of a Medicinal Plants' Mixture: A Case Report

**DOI:** 10.1155/crin/8207758

**Published:** 2024-12-19

**Authors:** Anfel Selles, Yanis Afir, Yasser Rahou, Lamis Debchi, Habiba Rafa-Debbah, Mohamed Rachid Bahriz, Ali Benziane

**Affiliations:** ^1^Department of Nephrology, CHU Bab El-Oued Hopital Mohamed Lamine Debaghine, Algiers, Algeria; ^2^Faculty of Medicine, Universite D'Alger 1, Algiers, Algeria

**Keywords:** acute kidney injury, *Artemisia absinthium*, *Centaurium erythraea*, *Marrubium vulgare*, tubulointertitial nephritis

## Abstract

Acute renal failure secondary to medicinal plants is common in countries where the use of traditional phytotherapy is preponderant. Although the nephrotoxic potentials of some herbal preparations have been well characterized, the use of many medicinal plants is still considered largely safe, often relying on weak evidence. Here, we report the case of a 17-year-old patient with severe acute renal failure, associated to an esophagitis with erosive gastritis as well as an inflammatory anemia, with no obvious etiology. After ruling out any other plausible explanation, the syndrome was attributed to the chronic intake of a mixture of three medicinal plants, previously unknown to be nephrotoxic: *Artemisia absinthium*, *Marrubium vulgare*, and *Centaurium erythraea*. A histological examination of a renal biopsy sample revealed an aspect of interstitial nephritis without antibody deposits. To our knowledge, this is the first reported case of acute kidney injury related to the consumption of these three plants and prompts further studies to carefully assess the safety of traditional medicinal products based on these plants.

## 1. Introduction

Traditional medicinal plants are widely used in many parts of the world including Algeria. Sellers advertise these plants as efficient treatments to various conditions ranging from functional disorders such as irritable bowel syndrome to diseases as serious as diabetes mellitus or cancer. In many cases however, preparation of such mixtures is insufficiently controlled, and insufficient pharmacovigilance makes it hard to track adverse events and toxicities related to these treatments.

Here, we present a case of a 17-year-old girl who presented an acute kidney injury (AKI) episode following the ingestion of a mixture of traditional medicinal plants.

## 2. Case Presentation

We report the case of a 17-year-old girl admitted in our clinic in February 2021 for an AKI episode likely related to the ingestion of an herbal tea mixture made of three common traditional medicinal plants used in Algeria: *Artemisia absinthium*, *Marrubium vulgare*, and *Centaurium erythraea*.

The patient had taken root extracts of the herbal mixture for the purpose of losing weight during 6 months prior to her hospital admission. She indeed reported a significant weight loss of 8 kg, associated however with severe fatigue, vomiting, and abdominal pain.

The patient's medical history does not reveal any particularity. She reported a history of an undocumented, and self-resolved, congenital heart defect, an iron deficiency anemia at the age of 7, successfully treated with iron supplementation, and a mild COVID-19 infection in January 2021. No surgical history was reported.

On general examination, the patient appeared alert, pale, and apyretic (temperature 37°C), with a blood pressure of 110/60 mmHg, a heart rate of 100 bpm, and a respiratory rate of 24 cpm. She was 167 cm tall, and her weight was 48 kg (BMI = 17 kg/m^2^).

The patient was nauseous, and the physical examination revealed signs of dehydration as well as lumbar pain. The abdomen was soft upon examination with an epigastric tenderness. Throat examination revealed a red and swollen throat.

Diuresis was quantified upon the patient's admission and was low (1 L/24 h). Urine dipstick test showed a microscopic hematuria (++), a proteinuria (++), and glycosuria (++) with normal glycemia.

The rest of the physical examination did not show any abnormality.

We performed blood test (Table 1) which revealed renal insufficiency (urea 13.1 mmol/L and creatinine 630 μmol/L), with hypokalemia (2.8 mmol/L), as well as microcytic hypochromic anemia of probable inflammatory origin (hemoglobin 9.6 g/dL with ferritin of 337 ng/L and an iron plasma level of 1.53 μmol/L) associated with mild leukopenia (3000 elements/mm^3^). No pathological hemolysis features were observed (negative direct Coombs test with a normal haptoglobin level). Besides, inflammatory markers were positive, namely, a positive CRP (55 mg/L) and a broad peak in the gamma band in the serum protein electrophoresis.

Liver (ALT, AST, ALP, GGT, bilirubin, and albumin), hemostatic (platelets, PT, APTT, and fibrinogen), and muscular (CPK and LDH) function tests were within the normal range.

We performed the biological test of the 24-h urine as well (Table 2), which showed for a diuresis of 700 mL, proteinuria (1.16 g/24 h) with ammonium urate crystals sediments, urinary creatinine of 7505 μmol/24 h, natriuria of 33 mmol/L, and kaliuria of 213 mmol/24 h. Urine culture did not show any signs of urinary tract infection later on.

The renal insufficiency appeared to be of acute onset as there were no signs of chronic kidney disease (CKD) on imaging or blood tests. On ultrasound examination, both kidneys were of normal size (119 × 45 mm for the right kidney and 94 × 50 mm for the left kidney) and anatomical position, with a preserved corticomedullary differentiation and without pyelocalyceal dilation. Besides, there were no biological signs of CKD: calcemia (26.25 mmoL/L), phosphoremia (1.26 mmoL/L), vitamin D (52 ng/L), and PTH (15 pg/L) as well as uric acid (279.6 μmol/L) were within the normal range.

Furthermore, the AKI episode was of probable organic and more specifically tubular origin given the presence of a natriuresis > 20 mm/L, *U*_Cratinine_/*P*_Creatinine_ < 40, *P*_Urea_/*P*_Creatinine_ < 50; *U*_Na_/*U*_*K*_ > 1; the presence of a significant proteinuria (1.02 g) with leucocyturia and ammonium urate crystals with a sterile UCBE, a high kaliuresis (contrasting with the hypokalemia), and finally a metabolic acidosis.

In addition to that, a hypermagnesemia was noted, without repercussion on heart rhythm (magnesuria analysis was not available). It was attributed either to an iatrogenic origin (intake of alginic acids) or to the metabolic acidosis.

To correct the renal function, the patient received intravenous rehydration at a rate of 3 L/24 h associated with a potassium supplementation. The evolution was somewhat favorable with an incomplete improvement of the renal function (creatinine 486 μmol/L).

Later, as the patient complained of persistent epigastric pain, an esophagogastroduodenoscopy was performed and revealed an aspect of peptic esophagitis associated with an antrofundic petechial and erosive gastritis of probable toxic origin. *Helicobacter pylori* eradication treatment was instituted nonetheless (quadritherapy: amoxicillin + metronidazole + clarithromycin + PPI [omeprazole]). The patient experienced a good evolution with an attenuation of the epigastric pain as well as the disappearance of the tonsilitis aspect on throat examination, indicating that it was likely related to the peptic esophagitis.

Regarding the AKI, the initial clinical and biological characteristics indicated a tubulointerstitial nephritis, with signs of proximal tubular involvement: hypokalemia with inadequate kaliuresis, metabolic acidosis, and a normoglycemic glycosuria. However, no origin of the AKI could be immediately identified.

We performed further investigations including autoimmunity screening (ANA and ANCA), tumor markers (AFP, CEA, CA 19–9, and CA 125), thyroid hormones, and vitamins B9 and B12 dosages. All these tests were negative or within the normal range.

**Figure 1:**
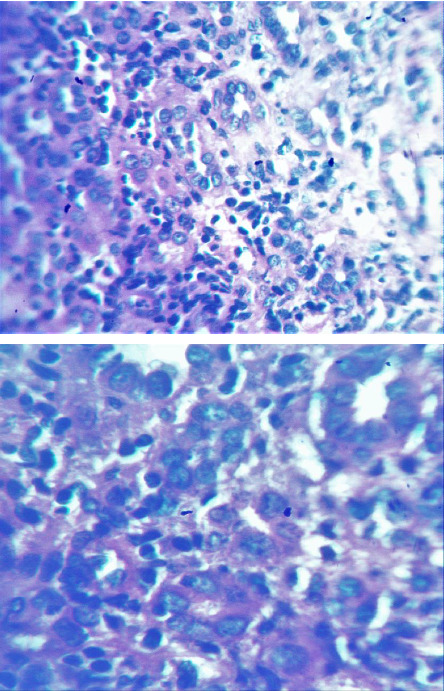
Histological slide from the patient's kidney biopsy, which show signs of nonspecific tubulointerstitial nephropathy.

We also performed a percutaneous ultrasound-guided kidney biopsy on the upper left renal pole. Results were in favor of a nonspecific tubulointerstitial nephropathy (shown in Figure 1).

Optical microscopy examination revealed the following: 4 glomeruli and 0 sclerosis glomerulus.

• On the tubulointerstitial compartment:
  • A reduction of tubular volume estimated at 40%
  • Acute tubular necrosis lesions by flattening of the epithelium of certain tubules with disappearance of the nuclei and accumulation of cellular debris and proteins inside the lumen
  • A lymphoplasmocytic inflammatory infiltrate in the interstitium (lymphocytes B), estimated at 20% and
  • An interstitial fibrosis of 15%
• No abnormalities were noted on the glomerular and vascular compartments.

Direct immunofluorescence examination of 29 glomeruli did not reveal any IgG or IgA, nor C3 and C1q complement fractions' deposits, with only traces of IgM.

Given these results, we started corticosteroid therapy: 0.5 mg/kg/day equivalent of prednisone for 1 month, followed by a progressive reduction over 6 months. Favorable clinical and biological evolutions were noted after 1 month of treatment, with a weight gain of 5 kg, a complete recovery of renal function with the following time course: Creatinine levels were at around 480 μmol/L at the beginning of the corticosteroid treatment, they fell to 122 μmol/L after 15 days of treatment and 70 μmol/L after 1 month. Creatinine remained stable after reduction and discontinuation of corticosteroids. In accordance, we observed a correction of the potassium serum level and the anemia (Hb 13 g/dL) and finally the disappearance of the glycosuria and proteinuria.

## 3. Discussion

Here, we described a case of a tubulointerstitial AKI associated to an inflammatory anemia and an erosive gastritis.

After thorough evaluation of the case and repeated examinations which ruled out other plausible cause, we believe that the only reasonable explanation of this AKI episode is linked to the medicinal plants that the patient was taking, namely, *A. absinthium*, *M. vulgare*, and *C. erythraea* mixture. This hypothesis was further confirmed by the favorable evolution and absence of recurrence after discontinuation of the incriminated mixture.

An interesting point is that during hospitalization, the patient received potential nephrotoxic drugs such as the PPI (omeprazole) [1] and amoxicillin [2], used for H. pylori's eradication, however both drugs were administered after the diagnosis of AKI was made. Similarly, the patient had a COVID infection in 2021. COVID can also cause impairment of the kidney function [3]. Nevertheless, in our case, it is unlikely to explain alone the AKI episode; it may however have acted as an aggravating factor making the kidney vulnerable to future injuries. Finally, dehydration secondary to vomiting triggered by the ingestion of the herbal mixture could result in acute tubular necrosis without intrinsic nephrotoxic effects of the plants *per se*. However, this is insufficient to explain the pathology results, with the observed nephritis. Furthermore, the renal function improved only slightly after rehydration and did not fully correct until the instauration of the corticosteroids therapy.

It should be noted that different weight loss herbal regimens are known to cause acute and CKDs. A notable example is the Balkan endemic nephropathy, caused by chronic intake of use of Aristolochia-based herbal remedies, which leads to poisoning with aristolochic acids [4]. In our case, however, the AKI episode does not seem to be related to Balkan nephropathy, as the patient ingested a preparation of different species. According to our literature search, we believe that this is the first published case of AKI related to the use of *A. absinthium*, *M. vulgare*, and *C. erythraea*. As explained below, the scientific literature does not attribute nephrotoxicity to any of these plants nor does it provide insights on the possible mechanisms by which they might cause tubular damage. Unfortunately, the herbal mixture itself was not available for us to analyze, and we could not get any information regarding its dosage and preparation. Toxicity screening was not available to perform for our patient as well.

Below we provide a summary of the medicinal properties and the toxic effects of each of the plants.

### 3.1. *A. absinthium*

*A. absinthium*, commonly called wormwood or *Chajrat Meriem* in Algeria [5], is a well-known plant whose beverage was popularized by artists and writers during the nineteenth century as a spirit drink [6]. It was used for centuries as a traditional remedy to treat various digestive, neurological, dermatological, or rheumatismal disorders. In some countries such as Germany, it is registered as an herbal drug, used against reduced appetite and weight loss [7]. Surprisingly, our patient used it for the exact opposite purpose.

Several in vitro results attribute possible therapeutic properties to wormwood extracts, such as antioxidant and immunomodulatory activities, or even antitumoral and antimicrobial effects [8].

Regarding the toxicity of *A. absinthium* extracts, the main concern is neurotoxicity, due to the presence of thujone, an aromatic hydrocarbon [6]. Long-term use of *A. absinthium's* essential oil is associated with a syndrome referred to as “Absinthism” which comprises various manifestations of central nervous system activation such as convulsions, hallucinations, and sleeplessness [8]. The main mechanism behind these manifestations seems to be related to the antagonist effect of thujone on GABA-dependent chloride channels, which results in an epileptic-like effect [6].

Other toxic effects of *A. absinthium* essential oils have been reported and include mental disorders, stomach cramps, vertigo, nausea, and vomiting and even mild chromosome aberrations associated with long-term exposure. Finally, absinthe may induce cholinergic effects through the blockage of acetylcholinesterase activity, leading to urine retention or chronic diarrhea [8].

Regarding nephrotoxicity, a case of AKI was reported by Weisbord et al. in a young man who had consumed woodwork essential oils [9]. However, according to the authors, the renal damage was most likely secondary rhabdomyolysis subsequent to seizures related to Absinthism. There was likely no direct renal damage in this case. In our case, however, nephrotoxicity appears to be a direct effect of the plants and no sign of rhabdomyolysis was present in our patient.

Besides, the same authors refer to an 1868 paper of R. Amory, who described a series of experiments on guinea pigs to which absinthe extracts were administered and which displayed evidence of kidney injury in the form of reddish urine appearance with albumin,and congested kidneys on pathological examination [10]. However, results of this experiment lack precision; there is no definitive evidence of direct kidney toxicity and may rather correspond to secondary renal involvement as a part of multiorgan dysfunction or secondary to rhabdomyolysis.

More recently, a study assessed the toxicity of continuous administration of absinthe to rats over 13 weeks. Overall, no toxicity was observed. Specifically, the study included a dosage of renal parameters and a pathological examination of the kidneys. Slight increases in blood urea nitrogen levels were reported, but these were considered by the authors to be of no toxicological significance. Histopathological examination showed basophilic tubules/calcifications of the kidney, although no emphasis was given to these observations by the authors, their significance remains unclear [11]. Finally, a 2020 study by Boudjelal et al. found no renal toxicity in animal models on acute administration of absinthe. The study included autopsy examination of the liver and kidneys of mice which revealed no pathological changes [5].

In definitive, we found no report in the literature of a direct nephrotoxic effect of *A. absinthium* in a human model. Interestingly, a study reports nephroprotective properties to absinthe extracts [7]. In this pilot uncontrolled clinical trial, *A. absinthium* supplementation had a positive antiproteinuric effect in patients with IgA nephropathy, probably due to the anti-inflammatory action of the plant [7]. Importantly, patients in this study were treated continuously with thujone-free wormwood extracts during 6 months, and no adverse effects or toxicity were reported.

### 3.2. *M. vulgare*

*M. vulgare*, commonly called *Marriout* in Algeria, is widely used as a therapeutic herb to treat various gastrointestinal and respiratory symptoms including asthma and dyspepsia [12].

Many beneficial effects are attributed to *M. vulgare*, including antioxidant, anti-inflammatory, antihypertensive, and antimicrobial activities [13, 14]. Here again, the plant is used against loss of appetite [12], despite our patient using it for the opposite purpose.

A report of the Committee on Herbal Medicinal Products (HMPC) of the European Medicines Agency does not indicate any undesirable effect linked to the plant [15], and a study of Elberry et al. did not find any sign of acute toxicity upon administration of *M. vulgare* extracts to mice at doses up to 1000 mg [16]. Similarly, a review of antidiabetic botanical herbs used in Algeria which included *M. vulgare* did not refer to any toxicity [17].

Nevertheless, a recent study seems to have identified possible hepatic toxicity linked to *M. vulgare* extracts, as they resulted in a high level of hepatic enzymes, which may be linked to the presence of a hepatotoxic furan ring [18]. Finally, a review of the phytochemical and pharmacological properties of *M. vulgare* reports an abortive effect possibly related to the presence of furanic labdane diterpene marrubin, and premarrubin, questioning its safe usage in pregnancy [13].

### 3.3. *C. erythraea*

*C. erythraea*, commonly called *Chiba* in Algeria, is also widely used as a therapeutic herbal medicine. Analgesic, antipyretic, diuretic, spasmolytic, and antimicrobial effects are attributed to this plant [19].

Regarding toxicology data, the study conducted by Tahraoui et al. on an animal model showed no signs of toxicity upon the oral administration of the whole plant; however, the intraperitoneal administration of lyophilized extracts increased mortality of mice starting from a dose of 8 g/kg [20]. Interestingly, this study also reports adverse effects of anorexia and weight loss, which may be in accordance with our case and the use of this herb as a mean to lose weight. Subchronical administration of the herb's extracts (during 90 days) did not result in perturbations of the kidney and liver function and showed no sign of toxicity in these organs on pathological examination either. The only notable biological effects were the decrease in glycemia and triglyceridemia levels. A study conducted on a rat model demonstrated a diuretic effect of *Centaurium erythraea* and an increase in electrolyte excretion [7]. The study also showed a delayed decrease in creatinine clearance which was attributed to a tubuloglomerular feedback mechanism related to the diuretic effect of the plant.

In definitive, we have found no evidence of nephrotoxicity attributed to *C. erythraea* in the literature.

## 4. Conclusion

In conclusion, we describe a case where our patient has continuously taken a combination of medicinal herbs during 6 months, which caused an episode of AKI with tubulointerstitial involvement, likely by direct and perhaps cumulative nephrotoxicity. Unfortunately, we are unable to fully characterize this toxicity nor to formulate hypotheses on its mechanisms due to lack of information regarding the dose of the herbal mixture and its exact composition.

We believe that this is the first published case of acute nephrotoxicity linked with the use of *A. absinthium*, *M. vulgare*, and *C. erythraea*. Further thorough toxicological studies should be encouraged to uncover possible toxicities of herbal plants in general, especially given their broad use in many parts of the world.

Finally, we believe that competent authorities should enforce stricter regulations and controls over these traditional remedies.

## Figures and Tables

**Table 1:** Blood tests' results.

Hemoglobin: 8.1 g/dL	Creatinine: 630 μmol/L	GGT: 20 U/L	LDH: 144 U/L
WBC: 2900 μL	Na+: 138 mmol/L	Total bilirubin: 3.77 mg/L	CRP: 55 mg/L
RBC: 3.24 10^6^ μL	K^+^: 2.8 mmol/L	ALT: 12 U/L	Cholesterol: 1.3 g/L
HCT: 27%	Phosphate: 1.26 mmol/L	AST: 15 U/L	Triglycerides: 1.56 g/L
MCV: 70 fL	Mg: 1.16 mmolx/L	Albumin: 39 g/L	Lipase: 80 U/L
Reticulocytes: 105.10^3^/mm^3^	Uric acid: 279.6 μmol/L	ALP: 60 U/L	Serum iron: 58 μg/L
Urea: 13.1 mmol/L	Calcium: 26.25 mmol/L	CK: 50 U/L	Ferritin: 337 ng/L
HCG: 0.2 mUI/L	TSH: 1.12 U/L	PTH: 15 pg/L	Vit D: 52 ng/mL
pH: 7.31	PCO_2_: 30 mmHg	PO_2_: 104 mmHg	HCO_3_: 19.7 mmHg
Haptoglobin: 1.5 g/L	HbA1c: 5.3%	Glycemia: 1.1 g/L	PT: 86% APTT: 30 s

**Table 2:** Urine tests' results.

Proteinuria: 1.05 g/24 h	Creatinine: 7505 mmol/24 h
Na^+^: 33 mmol/L	K^+^: 36 mmol/L

## Data Availability

Data sharing is not applicable to this article as no new data were created or analyzed in this study.
